# Supplementary material for: GPCR voltage dependence controls neuronal plasticity and behavior
Source: Nat Commun. 2021 Dec 13;12:7252. doi: 10.1038/s41467-021-27593-x (PMC8668892; doi:10.1038/s41467-021-27593-x)
Supplement: Supplementary file 1 — Supplementary Information [file 41467_2021_27593_MOESM1_ESM.pdf]

Supplementary information

GPCR Voltage Dependence Controls Neuronal Plasticity and Behavior

Eyal Rozenfeld[1,2], Merav Tauber[3], Yair Ben-Chaim[3], Moshe Parnas[1,2*]

Affiliations:

[1] Department of Physiology and Pharmacology, Sackler School of Medicine, Tel Aviv University, Tel Aviv 69978, Israel

[2] Sagol School of Neuroscience, Tel Aviv University, Tel Aviv 69978, Israel

[3] Department of Natural and Life Sciences, The Open University of Israel, Ra'anana 43107, Israel

*Corresponding author. Email: mparnas@tauex.tau.ac.il.

Contents: Supplemental Figures 1-6 and supplementary methods

A

M1	101	LALDYVASN	376	TWTPYNIMV	400	WELGYWLCY
M2	99	LALDYVVSN	398	TWAPYNVMV	422	WTIGYWLCY
M3	144	LALDYVASN	502	TWTPYNIMV	526	WNLGYWLCY
M4	108	LALDYVVSN	411	TWTPYNVMV	435	WSIGYWLCY
M5	106	LALDYVASN	453	TWTPYNIMV	477	WHLGYWLCY
mAChR-A	180	LALDYLASN	728	TWTPYNILV	754	WDFFYALCY
mAChR-B	209	LSVDYTVCL	689	CWTPYHVLA	715	YMFSYFLCY

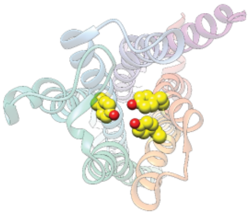

B

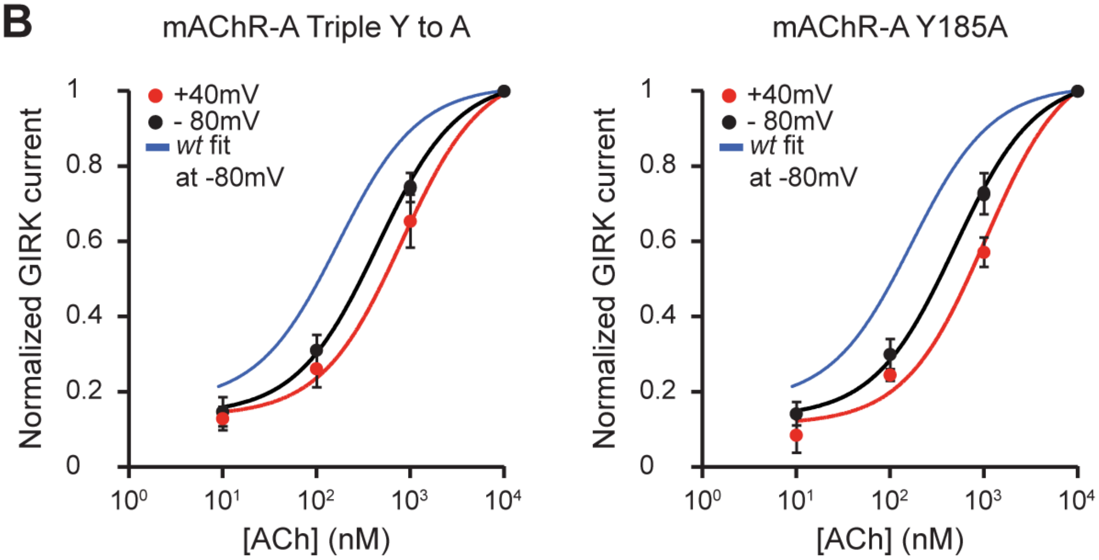

C

M1	221	ELAAL
M2	218	KKDKK
mAChR-A	300	KDLPN
mAChR-A-KK	300	KKLKN

Supplemental Figure 1: Amino acids involved in mAChR-A voltage dependence

A. *Left*, multiple sequence alignment of the muscarinic receptors of mice, mAChR-A and mAChR-B. The three tyrosine residues (marked with a brown background), which constitute the voltage sensor of mouse M_1R and M_2R, are conserved in flies. *Right*, a top view of M_2R. The three tyrosine residues are labeled in yellow. Protein structures were generated using UCSF chimera [1]

B. Dose-response curves for mAChR triple Y to A (left; n=11 at -80 mV and 9 at +40 V) and mAChR Y185A (right; n=11 at -80 mV and 2 at +40 V) evoked GIRK current in oocytes at -80 mV (black) and +40 mV (red). A decline in activity is observed in both cases. Error bars represent the standard error of the mean (SEM).

C. Multiple sequence alignment of the muscarinic receptors N-terminal region of the third intracellular loop that affects voltage dependency.

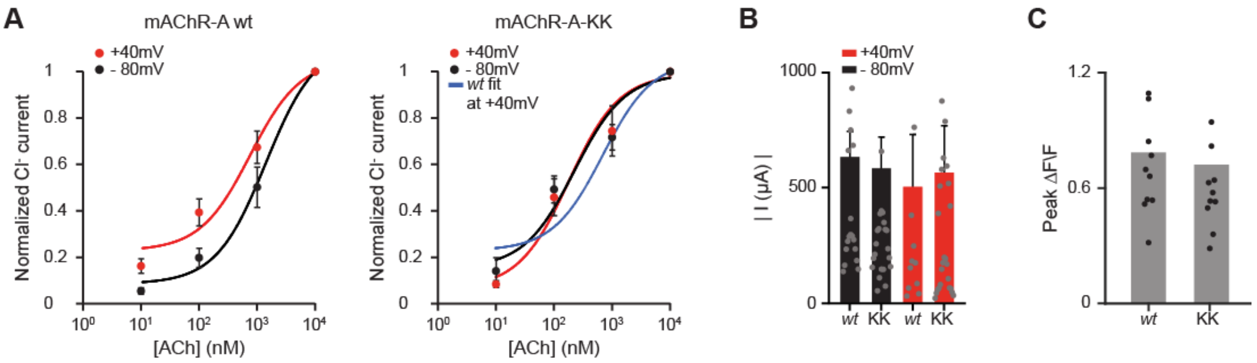

Supplemental Figure 2: mAChR-A G_q pathway is functional and voltage dependent

A. Dose-response curves for *wt* mAChR-A (left) and mAChR-A-KK (right) G_q activated Cl^- currents in oocytes at -80 mV (black; n=10,11,6,26 and 7,10,10,28 for *wt* mAChR-A and mAChR-A-KK, respectively and in ascending order of ACh nM) and +40 mV (red; n=9,15,16,24 and n=6,7,7,20 for *wt* mAChR-A and mAChR-A-KK, respectively and in ascending order of ACh nM). As with the GIRK currents, mAChR-A-KK is in the high activity state as indicated by the overlap with the dose response curve of *wt* mAChR-A at +40 mV (blue line)

B. Absolute current values of *wt* mAChR-A and mAChR-A-KK, for ACh evoked currents in oocytes at -80 mV (left, black; n=24 and 29, respectively) and +40 mV (right, red; n=13 and 29, respectively) elicited by a saturating level (10^4 nM) of ACh. No difference is observed between the different conditions, indicating that the receptor maximal activity is not impaired.

C. 2-photon functional imaging was performed using GH298-GAL and the genetically encoded Ca^{2+} indicator UAS-GCaMP6f on the background of *wt* or mAChR-A-KK flies. To examine only Ca^{2+} release from internal stores which results from the activation of the G_q pathway, nominal 0 Ca^{2+} external solution with TTX was used. A single plane of the AL was imaged. Peak fluorescence signal for *wt* mAChR-A (left, n=11) and mAChR-A-KK (right, n=12) elicited by a saturating level of muscarine (10^5 nM) are presented. No significant difference is observed, indicating that the release of Ca^{2+} from internal stores by the G_q pathway is not impaired. Each dot represent a single fly.

For all panels, error bars represent the standard error of the mean (SEM).

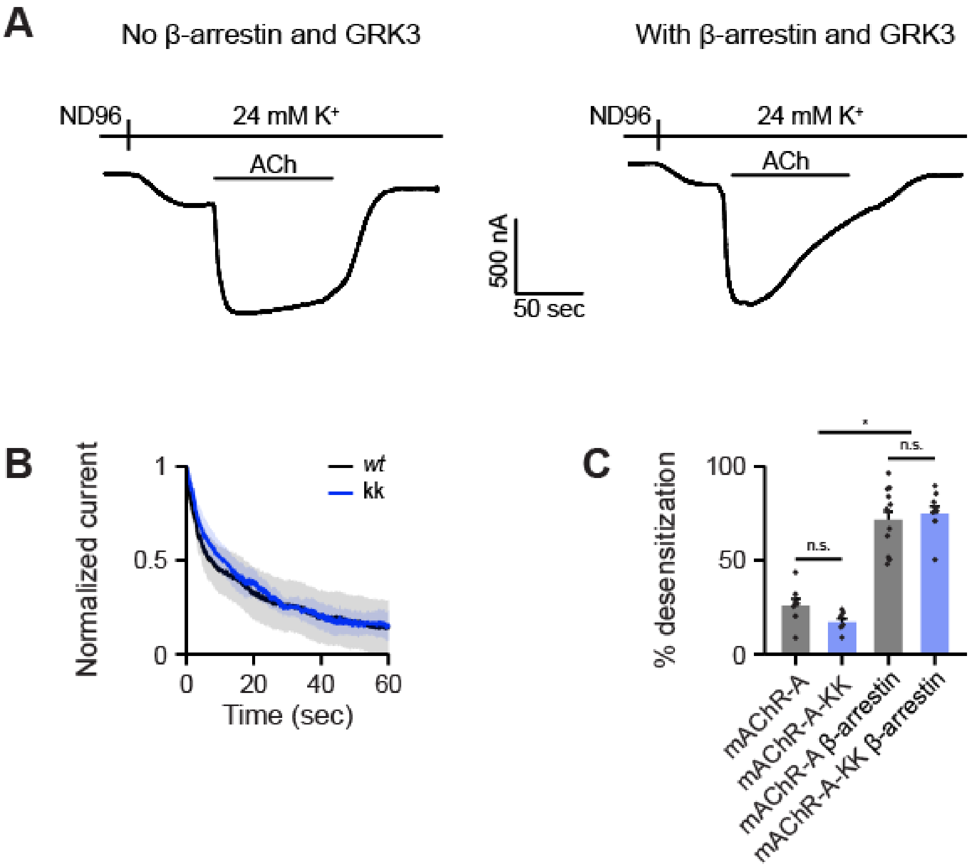

Supplemental Figure 3: mAChR-A-KK mutant does not affect β-arrestin signaling

A. Example of traces showing receptor desensitization by β-arrestin as measured from ACh-evoked GIRK currents decay. Weak desensitization of ACh-evoked GIRK currents is observed when the mAChR-A receptor was expressed alone (left), and strong desensitization when mAChR-A was co-expressed with β-arrestin and GRK3 (right).

B. Averaged traces depicting the desensitization kinetics with β-arrestin and GRK3 in *wt* mAChR-A (black line; n=7; SEM is shaded area) and in mAChR-A-KK (blue; n=6; SEM is shaded area). The currents are normalized to the maximal current evoked by ACh at a given oocyte.

C. The mean desensitization values obtained from traces such as shown in panel A. No significant difference was observed for both conditions (i.e. with or without β-arrestin and GRK3) between *wt* mAChR-A (grey; n=7 and 12 respectively) and mAChR-A-KK (blue; n=6 and 8 respectively), ($p = 0.013$, Two sample two sided *t*-test). Error bars represent the SEM.

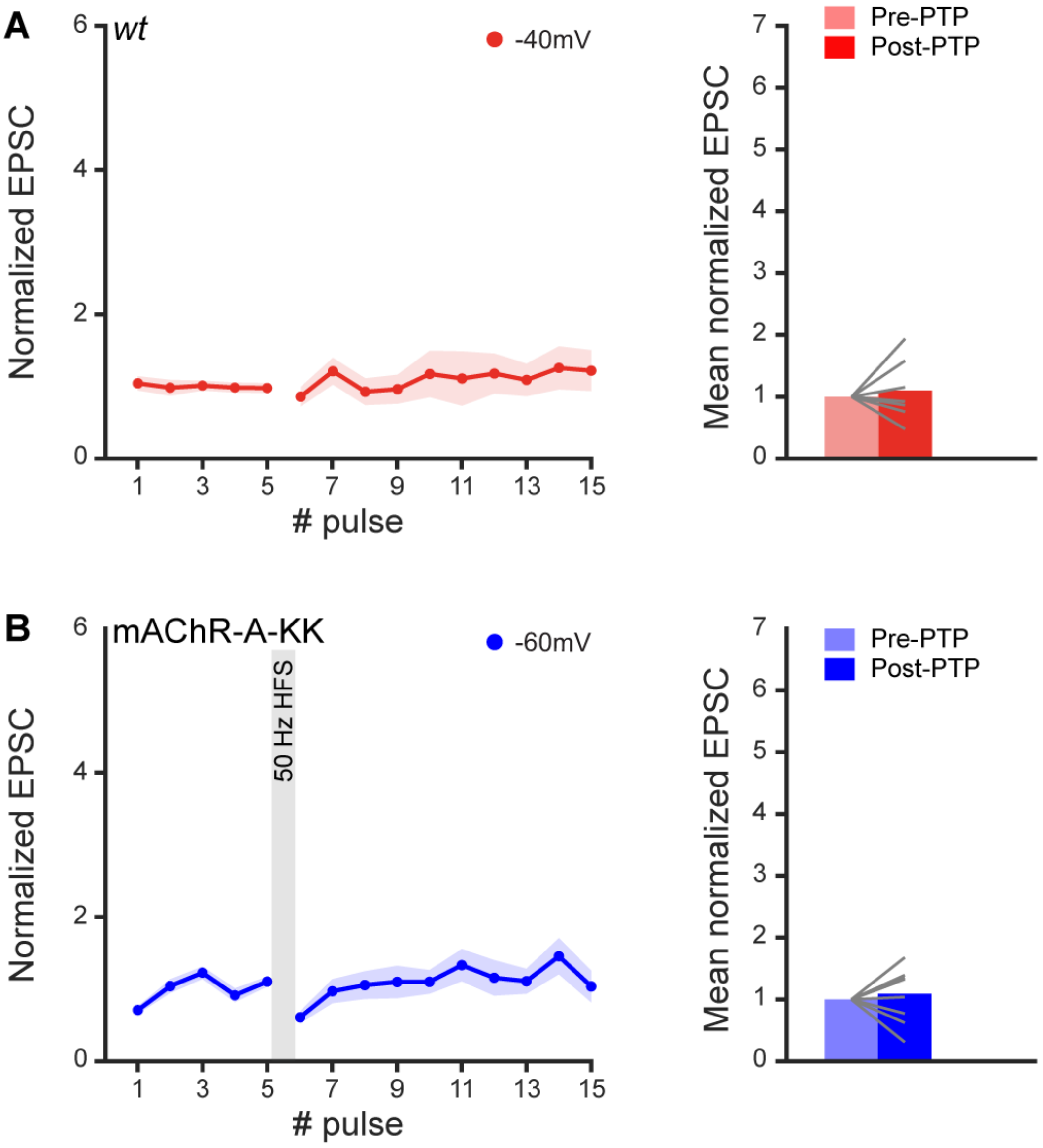

Supplemental Figure 4: Voltage step and lower stimulation frequency controls for PTP

A. *Left*, normalized EPSC before and after depolarization in *wt* flies (expressing *wt* mAChR-A) for a holding potential of -40 mV. *Right*, mean normalized EPSC obtained from the data presented on the left. Applying a voltage step of -40mV without the HFS protocol did not change iLN EPSC (n=7, Paired sample *t*-test).

B. *Left*, normalized EPSC before and after a lower frequency PTP (50 Hz) obtained in the mAChR-A-KK fly strain (expressing mAChR-A-KK) for holding potential of -60 mV. *Right*, mean normalized EPSC obtained from the data presented on the left. No potentiation was observed. GH298-GAL4 was used to drive UAS-GFP. Each line represents a single fly (n=9, Paired sample *t*-test).

For all panels, shaded error bands represent the standard error of the mean (SEM).

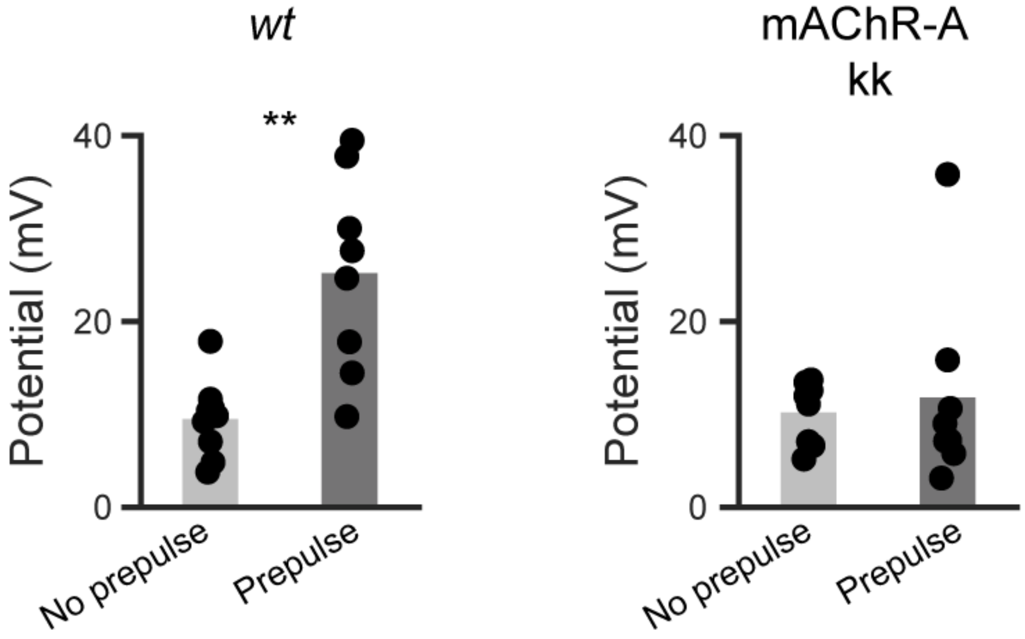

Supplemental Figure 5: Voltage pre-pulse increases mAChR-A responses

A depolarizing pre-pulse step (40 mV for 30 seconds) before the muscarinic pulse potentiated the response to muscarine in *wt* flies (*left*, no pre-pulse, n=9, pre-pulse, n=8) but not in the mAChR-A-KK fly strain (*right*, no pre-pulse, n=8, pre-pulse, n=8). Each dot represents a single fly (p(wt) = 0.0009, Two sample two sided *t*-test).

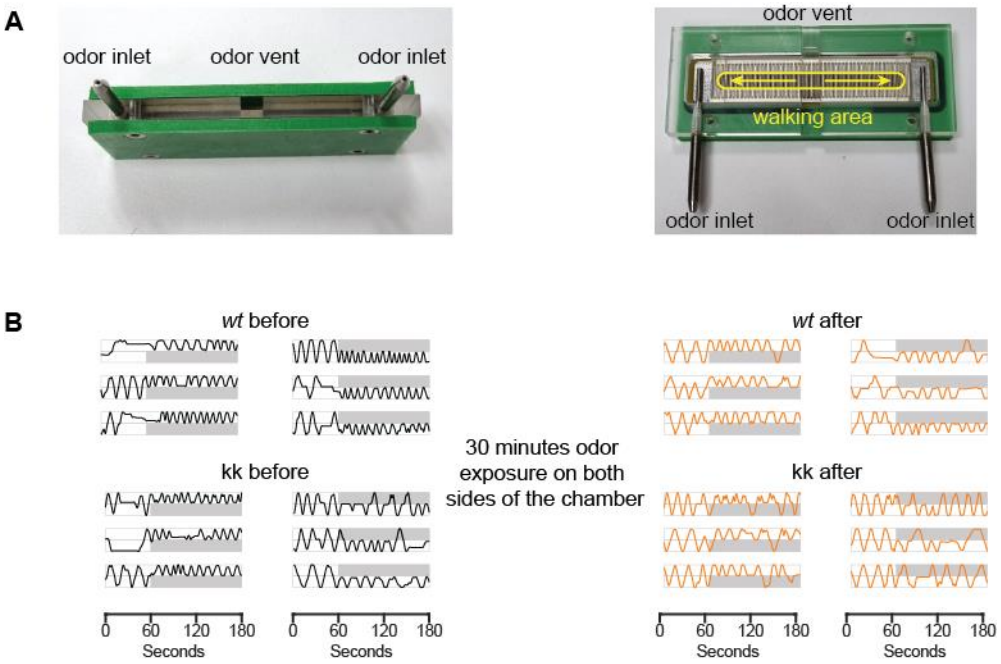

Supplemental Figure 6: Behavior apparatus

A. A photograph (*left*) of the behavioral chamber used in the odor habituation experiments. Odor flows in from both sides via the odor inlets and is vented by an opening in the middle of the chamber. The chamber hold a single fly and 20 chambers are used for each experimental session. The fly walking area (*right*, top panel removed) is constrained by the two green panels limiting flies to movement to the x axis.

B. Examples of flies walking trajectories inside the behavioral chamber and scheme of the habituation protocol. At the beginning of the experiment no odor is presented for one minute, then an odor is presented to the right side of the chamber (gray) for two minutes. This protocol is then repeated for with the odor presented to the left side of the chamber. Following this, the odor is presented in both sides for thirty minutes. Finally, the initial protocol is repeated. *wt* flies retain their odor avoidance after thirty minutes of odor exposure whereas mAChR-A-KK show reduced odor avoidance.

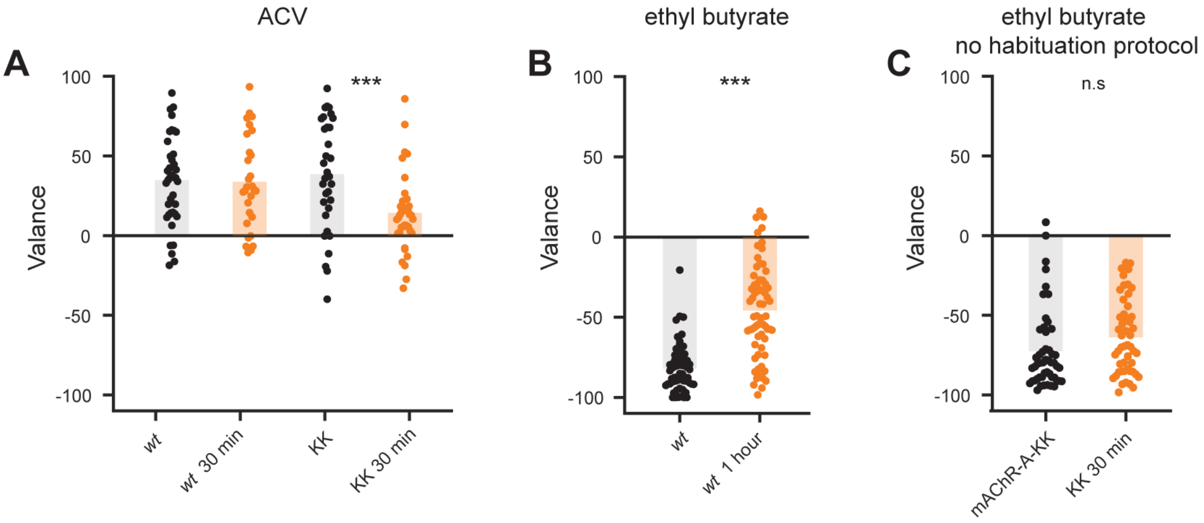

Supplemental Figure 7: Controls for behavioral habituation

A. The valence (positive values indicate attraction) flies assign to apple cider vinegar (ACV) before and after the odor habituation protocol (30 minute exposure). For *wt* flies, there was no change in odor valence, however, the mAChR-A-KK fly strain displayed a strong and significant reduction in odor attraction, indicating odor habituation. (*wt* before, n=39, *wt* after, n=29, mAChR-A-KK before n=33, mAChR-A-KK after n= 38, p(kk) = 0.0009, Two sample two sided *t*-test). Each dot represent a single fly.

B. The valence *wt* flies assign to ethyl butyrate before and after the odor habituation protocol (1 h exposure). A strong and significant reduction in odor avoidance was observed indicating odor habituation. (*wt* before, n=65, *wt* after, exposure n=66, $p<10^{-10}$, Two sample two sided *t*-test). Each dot represent a single fly.

C. The valence mAChR-A-KK flies assign to ethyl butyrate before and after 30 minutes without exposure to the odor. No significant reduction in odor avoidance was observed. (mAChR-A-KK before, n=48, mAChR-A-KK, after 30 min n=45, Two sample *t*-test). Each dot represent a single fly.

Supplementary Materials and Methods

Generation of mAChR_D301KP303K (mAChR-A-KK)

Design of gRNA

Two guide RNA sequences, CCGTGCCGAAGGTGATGTACTGG and tatatatttagaccgaccaaagg, were selected as CRISPR/Cas9 targets. A donor template (see sequence below) was designed to perform the CRISPR/Cas9 mediated HDR (homology directed repair) to introduce D301KP303K double mutations into mAChR allele.

Donor template sequence (w/o backbone)

GGTGCAGCGAAAAGGATAAGCAGTGGATGAGGCTCAACTAAGAGACAAGCCAAAC
ACAGAGAGAGAGAGAGAGAGAAAGCTAACGACAGCGAGCATCGTAATCCATACAGCT
GCAAAGTAGATAAAATCACGTATCCGTCATGGAGCCGGTCATGAGTCTGGCATTGG
CCGCCCATGGACCGCCTAGCATACTAGAGCCGCTGTTTAAGACCGTAACCACGAGC
ACAACGACCACCACCACCACGACGACCAGCACGACGACGACGACGGCGAGTCCTGC
AGGGTATTCGCCAGGATACCCGGGCACCACTCTTCTGACCGCCCTGTTCGAGAACCT
GACGTCCACGGCGGCGAGCGGGCTATACGATCCCTATTCGGGAATGTACGGAAACC
AGACGAACGGCACCATCGGCTTCGAGACAAAGGGACCTCGGTACTCACTGGCCTCC
ATGGTGGTCATGGGCTTCGTGGCGGCGATTTTGAGCACGGTGACGGTGGCGGGCAA
TGTCATGGTGATGATATCCTTTAAGATCGACAAGCAGCTGCAGACGATCAGCAACTA
CTTCTTGTTCTCGCTGGCCATCGCGGACTTCGCCATTGGCGCTATATCGATGCCGCTT
TTTGCGGTGACCACAATCCTGGGCTACTGGCCCTTGGGTCCCATCGTCTGCGACACC
TGGCTGGCCCTGGACTACCTGGCATCGAATGCTTCAGTGCTGAACCTGCTGATCATC
AGTTTCGATCGGTACTTCAGTGTCACTCGACCGCTGACGTACCGCGCCAAAAGGACC
ACCAACCGGGCGGCGGTGATGATTGGCGCCGCCTGGGGCATCAGCCTGCTCCTCTG
GCCGCCCTGGATCTACAGCTGGCCCTACATCGAGGGCAAGCGGACGGTGCCCAAGG
ACGAGTGCTACATCCAGTTCATCGAGACCAATCAGTACATCACCTTCGGCACGGCAC
TGGCCGCCTTCTACTTCCCGGTCACCATCATGTGCTTCCTCTACTGGCGCATCTGGCG
CGAGACGAAAAAGCGGCAGAAGAAGCTGAAGAACCTGCAGGCGGGCAAGAAAGAC
TCCAGCAAGAGATCAAACAGCAGgtgaggcataagataagtgtatacagtaagatctcttgaagaattactttagtttca
gtaataccttaaattaatattaaattatttaacgttgcgctgaaatatcttgttcttatttggcgactcaatgagctgcatcattttaaatttgaaatgta
gaaacagttcatttcgcagcttatcaattcgttttgtttgattttatgtcacgttgaaacgggcatgattacagtcgaagActttggtcggtctaaat
atataactaagcatttgaacaaataatcttattttgtgatgtttgcagTGACGAAAACACGGTGGTGAATCACGCCT
CTGGCGGCCTGCTGGCCTTCGCCCAGGTGGGCGGAAACGACCATGACACCTGGCGA
CGTCCGCGCTCCGAGAGTTCACCGGACGCGGAGAGTGTCTACATGACCAACATGGT
CATCGACTCCGGCTACCACGGGATGCACTCGCGCAAGTCAAGCgtaggacactctgaccgcctttt
ccctgttcctggttactaattcccactatccacagATCAAAAGCACGAATACAATCAAAAAATCGTACACC
TGCTTCGGCAGCATCAAGGAGTGGTGCATTGCGTGGTGGCACTCCGGTCGCGAGGA
CTCCGACGACTTCGCCTACGAGCAGGAGGAGCCTTCTGATTTAGGGTATGCAACACC
AGTAACAATTGAAACTCCTTTACAAAGCTCCGTCTCCAGgtagtaaaaacaatcaaaattctgaacttta
atcgtctatatttcgagtaagccgacaccgcacccactctctcaccccaagttatatgcgtacgactcgtacgacttccattgcacccattgcac
ttttccgagagccaaacaaacaacctgcacccaaaacacatatatcgattcgatattccgtccaatgtctgtgcacacaaacgcaaagtggca
gtggtgaaaagtgtccaggaggatgagtttggtcccattctccactggccaccacgatgcaccaatccctttccctctcccttgaccattccca

atcctccttccacacacgaatccatcaaagtcatagcatccagcactaaaataatccacgtagatggaaggctttgcaccactgcacacatcgggatggaaactaattgaggcttatagtccaatgtagttggagcaggacattgtactccggagtttgttgattccgatg

In addition to designed amino acid changes (in red text) for D301K/P303K, two extra point mutations were designed at the PAM region of gRNA targets (highlighted in green) in order to prevent the CRISPR/Cas9 system from attacking the established mutant allele. Since the first gRNA target falls into an exon (capital letters), a silent mutation (C-T) was designed to keep D265 intact. Two homologous arm sequences were underlined.

Cloning procedure

Cloning of gRNA expression was done with the pCFD3 plasmid. Two pairs of oligos for two gRNAs are listed below.

Oligo pair for gRNA1 GTCGCCGTGCCGAAGGTGATGTAC/ AAACGTACATCACCTTCGGCACGG

Oligo pair for gRNA2 GTCGtatatatttagaccgaccaa/AAACttggtcggtctaaatatata

The donor plasmid was built through a three-piece DNA assembly into vector backbone pUC57-Kan with two PCR products and one synthesized DNA fragment.

Piece#	Method	PCR primers / synthesized DNA sequence
1. (left arm)	PCR	tacacGGTCTCaTAGCGGTGCAGCGAAAAGGATAAGCAG
		gatcaGGTCTCaCTGATTGGTCTCGATGAACTGGATGTAGCAC
2. (mutant allele)	DNA synthesis	A 426 base pair DNA fragment containing all designed mutations was directly synthesized (by IDT). tacacGGTCTCaTCAGTACATCACCTTCGGCACGGCACTGGCCGCCTTCTACTTCCCGGTCACCATCATGTGCTTCCTCTACTGGCGCATCTGGCGCGAGACGAAAAAGCGGCAGAAGAAGCTGAAGAACCTGCAGGCGGGCAAGAAAGACTCCAGCAAGAGATCAAACAGCAGgtgaggcataagataagtgtatacagtaagatctcttgaagaattactttagtttcagtaataccttaaattaatattaaattatttaacgttgcgctgaaatatcttgttcttatttggcgactcaatgagctgcatcattttaaatttgaaatgtagaaacagttcatttcgcagcttatcaattcgttttgtttgattttatgtcacgttgaaacgggcatgattacagtcgaagactGAGACCtcagt
3. (right arm)	PCR	tacacGGTCTCaagactttggtcggtctaaatatataactaagcatttg
		gatcaGGTCTCaATCGcatcggaatcaacaaactccggag
Ligation (donor plasmid)	Golden Gate Assembly	Ligating #1,2 & 3 into pUC57-kan vector by BsaI/T4 ligase cocktail via their cohesive ends (highlighted in green).

Injection, screening and balancing

Plasmids (prepared with plasmid midi kit) for two gRNAs and a donor were mixed at their respective concentrations (50 ng/ul for each gRNA and 120 ng/ul for donor) and injected into 720 embryos of Bloomington stock #54591 (y[1] M{w[+mC]=nos-Cas9.P}ZH-2A w[*]) with the 2nd chromosome isogenized.

From the progenies of G0 males X BcE/CyO crosses, 96 F1 males with CyO phenotype were randomly selected to set up individual F1 crosses with BcE/CyO virgin females. Founder F1 males were then sacrificed and single fly extracts were made for screening for the existence of the mutant allele. A pair of PCR primers, CAATCCTGGGCTACTGGCC and caaataagaacaagatatttcagcgcaacg flanking the double mutations, were used to amplify a 382 bp PCR fragment and PCR products were treated with an Exonuclease/Phasphotase cocktail before sequencing analyses using one of the end primers.

Final independent lines were established from sequence-confirmed F1 mutant males (one line/positive independent G0). Genomic PCR followed by sequencing was performed on F2s from each balanced line to further confirm the existence of the mutant allele.

Molecular characterization

Individual F2/F3 were subject to genotyping PCR using single fly extracts as template.

1. We picked a single fly from each vial and made fly extract by crushing the insect with 50 μl squishing buffer (10 mM Tris-Cl, pH8.2, 1 mM EDTA, 25 mM NaCl and 200 ng/μl freshly prepared Proteinase K). We incubated the lysate at 37 °C for 30 minutes and then heat at 95 °C for 5 minutes.

2. In addition to previously mentioned PCR product made to verify the mutant allele, two more PCR reactions were performed using two sets of primers (see table below). Each set contains a mutation specific primer and a primer located up or downstream of homologous arm sequences. PCR products were gel purified and sequenced using the same end primers. The alignment of three overlapping PCR fragments would allow us to confirm correctness of the entire sequence involved in crispr/HDR process.

	Primer sequence	Product size (ca.)
Set 1	cggtcggaccgtttacatatc (upstream of crispr/HDR region)	1.1 kb
	GCCTGCAGGTTCTTCAGCTT (mutation specific)	
Set 2	AGCGGCAGAAGAAGCTGAAG (mutation specific)	1.4 kb
	ccagaaatgtctcaatgctcgtagag (downstream of crispr/HDR region)	

References

1. Pettersen, E. F. *et al.* UCSF Chimera - A visualization system for exploratory research and analysis. *J. Comput. Chem.* **25**, 1605–1612 (2004).
